# Mitochondrial Lipid Metabolism Genes as Diagnostic and Prognostic Indicators in Hepatocellular Carcinoma

**DOI:** 10.2174/1389202924666230914110649

**Published:** 2023-10-27

**Authors:** Xuejing Li, Ying Tan, Bihan Liu, Houtian Guo, Yongjian Zhou, Jianhui Yuan, Feng Wang

**Affiliations:** 1 Department of Physiology, School of Basic Medical Sciences, Guangxi Medical University, Nanning, China;; 2 Research Center for Biomedical Photonics, Institute of Life Science, Guangxi Medical University, Nanning, China;; 3 Key Laboratory of Biological Molecular Medicine Research, Guangxi Medical University, Education Department of Guangxi Zhuang Autonomous Region, Nanning, China;; 4 Department of Biochemistry and Molecular Biology, School of Basic Medical Sciences, Guangxi Medical University, Nanning, China

**Keywords:** Hepatocellular carcinoma, mitochondria, diagnosis, prognosis, immune, biomarker

## Abstract

**Background:**

Due to the heterogeneity of Hepatocellular carcinoma (HCC), there is an urgent need for reliable diagnosis and prognosis. Mitochondria-mediated abnormal lipid metabolism affects the occurrence and progression of HCC.

**Objective:**

This study aims to investigate the potential of mitochondrial lipid metabolism (MTLM) genes as diagnostic and independent prognostic biomarkers for HCC.

**Methods:**

MTLM genes were screened from the Gene Expression Omnibus (GEO) and Gene Set Enrichment Analysis (GSEA) databases, followed by an evaluation of their diagnostic values in both The Cancer Genome Atlas Program (TCGA) and the Affiliated Cancer Hospital of Guangxi Medical University (GXMU) cohort. The TCGA dataset was utilized to construct a gene signature and investigate the prognostic significance, immune infiltration, and copy number alterations. The validity of the prognostic signature was confirmed through GEO, International Cancer Genome Consortium (ICGC), and GXMU cohorts.

**Results:**

The diagnostic receiver operating characteristic (ROC) curve revealed that eight MTLM genes have excellent diagnostic of HCC. A prognostic signature comprising 5 MTLM genes with robust predictive value was constructed using the lasso regression algorithm based on TCGA data. The results of the Stepwise regression model showed that the combination of signature and routine clinical parameters had a higher area under the curve (AUC) compared to a single risk score. Further, a nomogram was constructed to predict the survival probability of HCC, and the calibration curves demonstrated a perfect predictive ability. Finally, the risk score also unveiled the different immune and mutation statuses between the two different risk groups.

**Conclusion:**

MTLT-related genes may serve as diagnostic and prognostic biomarkers for HCC as well as novel therapeutic targets, which may be beneficial for facilitating further understanding the molecular pathogenesis and providing potential therapeutic strategies for HCC.

## 1 INTRODUCTION

Primary liver cancer ranks as the sixth most prevalent form of cancer worldwide, with an estimated 905,677 new cases and 830,000 fatalities in 2020, and is the third leading cause of cancer-related mortality on a global scale [1]. Hepatocellular carcinoma (HCC) is the predominant form of primary liver cancer, accounting for 75-85% of all cases [1, 2]. It typically arises in the setting of chronic liver disease and carries a poor prognosis due to its aggressive nature [3]. Only a small percentage of patients, ranging from 5% to 15%, meet the criteria for surgical removal [4, 5]. This option is only suitable for those in the early stages of the disease. Unfortunately, most patients are not diagnosed until their tumor has progressed to an advanced stage, causing them to miss out on the optimal window for treatment [6]. Therefore, it is imperative to identify effective diagnostic and prognostic biomarkers for HCC that can facilitate early and precise interventions, ultimately leading to improved outcomes for patients with this disease.

Lipid homeostasis in mitochondria is essential in maintaining functional mitochondria, and it relies on the balance between mitochondrial lipids synthesis and redistribution to respond to different metabolic conditions [7]. Accumulating evidence had demonstrated that reprogramming of lipid metabolism was a new hallmark of malignancy, which was particularly crucial in cancer [8]. Cancer cells exploit the manipulation of mitochondrial lipid metabolism (MTLM) to optimize survival, proliferation, and drug resistance, resulting a well-established adaptive response that drives an aggressive phenotype [9]. Lipid metabolism pathways have emerged as a promising anticancer strategy, yet the diagnostic and prognosis significance of MTLM genes in HCC remains unexplored.

In this study, dysregulated expression of MTLM genes was identified through analysis of the GEO (Gene Expression Omnibus) database. The diagnostic value was analysed in the TCGA (The Cancer Genome Atlas) database and the Affiliated Cancer Hospital of Guangxi Medical University dataset (GXMU cohort). An independent prognostic risk score model was constructed after LASSO-Cox analysis based on the TCGA database, and it was externally validated with a public database and GXMU cohort. A nomogram was constructed by integrating clinical data and the prognostic gene signature to predict the survival probability of patients with HCC. Finally, the study also evaluated the status of immunity and genomic mutations. Our primary objective was to conduct a comprehensive bioinformatic analysis, integrating gene expression profiling from TCGA, GEO, ICGC and GXMU with clinical characteristics, to identify MTLM genes associated with HCC diagnosis, prognosis and therapeutic targets.

## 2 MATERIALS AND METHODS

### 2.1 Samples and Transcriptome Sequencing

Paired tumor and adjacent non-tumor tissues were obtained from 116 patients with hepatocellular carcinoma who underwent surgical resection at the Affiliated Cancer Hospital of Guangxi Medical University, which served as the validation set. The Human Subject Research Ethics Committee of Guangxi Medical University (Ethical Number: 20200137) approved this study. The experimental methods were conducted in accordance with relevant guidelines and regulations. Detailed clinical and pathological data are shown in Table **1**. Each sample underwent RNA extraction and strand-specific RNA-seq library preparation. The RNA was purified, followed by cDNA synthesis, cDNA library preparation and sequencing conducted by WUXI NextCODE in Shanghai. Quality management was performed using Fast QC and clean reads were mapped to the human genome (hg19) using HiSeq2 and StringTie prior to bioinformatics analysis [10].

### 2.2 Public Data Collection and Identification of Differentially Expressed Genes (DEGs)

Three hundred eighty samples from three microarray datasets (GSE84402, GSE101685, and GSE102079) on the GPL570 platform were analyzed for differentially expressed genes (DEGs) analysis using the “limma” package in R [11]. RNA expression and clinical data for 50 normal and 369 tumor samples with Liver hepatocellular carcinoma (LIHC) were retrieved from the TCGA database as a testing cohort. The validation cohorts included 221 LIHC patients from GSE14520_GPL3921 and 230 LIHC patients from ICGC (International Cancer Genome Consortium).

### 2.3 Extraction of Hub Genes in MTLM

A systematic approach was employed to search for gene sets related to lipid metabolism in the Molecular Signatures Database v7.5.1 (MSigDB; http://www.gsea-msigdb.org/gsea/msigdb/search.jsp). The DEGs associated with lipid metabolism were submitted to the STRING online database (http://string-db.org) for identification of interacting genes. The Cytoscape software (v3.9.0) was utilized to visualize the protein-protein interaction (PPI) networks, and the Molecular Complex Detection (MCODE) clustering algorithm identified hub genes in the network. The regions were defined based on the following criteria: degree cutoff of 5, node score cutoff of 0.2, max depth of 100, and K-score of 5. Gene ontology (GO) analysis was performed using the “clusterProfiler” package [12] in R with a significance level threshold criterion set at *p* < 0.05.

### 2.4 Protein Level and Kaplan Meier Plotter

The Human Protein Atlas (HPA) (https://www.protein atlas.org/) was utilized to investigate the protein-level expression associated with MTLM genes. The Kaplan-Meier (KM) survival analysis plot was generated using the “survival” R package.

### 2.5 Gene Signature Identification and Risk Score Construction

Univariate Cox regression analysis identified MTLM genes with potential prognostic value for overall survival (OS). To expand the potential gene pool, a cut-off value of *P* < 0.2 was utilized for subsequent analysis. The “glmnet” package in R was used with the least absolute shrinkage and selection operator (LASSO) regression to select linear models and retain important variables [13]. The risk score was established by utilizing gene expression and corresponding coefficients according to the following formula.


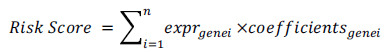


The prognostic risk characteristics were assessed using “survival” and “timeROC” R packages. The LASSO regression and subsequent analyses were performed based on 224 samples with complete clinical data (including age, gender, sex, tumor grade, tumor stage, OS status and time information) in TCGA.

### 2.6 Construction and Verification of a Nomogram

Univariate and multivariate Cox regression analyses were conducted to validate the independent prognostic factors using complete clinicopathological factors from TCGA (including stage, TNM stage, gender, age, and grade) and GXMU cohorts (gender, age, Barcelona Clinical Liver Cancer System (BCLC) classification system, Edmonson classification system, and recurrence status). The patients’ characteristics were succinctly delineated in Table **1**. Additionally, a stepwise Cox regression model was employed to investigate the predictive efficacy of combining risk score and clinical characteristics from TCGA cohort [14]. Subsequently, a nomogram was constructed using the “survival” and “rms” packages in R based on the stepwise variable Cox analysis. The performance of nomogram was evaluated by calculating the C-index and plotting the area under the decision curve analysis (DCA) [15]

### 2.7 Single-sample Gene Set Enrichment Analysis (ssGSEA) and Tumor Mutation Burden(TMB) Estimation

The ssGSEA method from the R package GSVA [16] was used to assess immune cell infiltration based on gene expression levels of 28 published gene sets [17]. Maftools [18] was employed to identify and visualize the top 20 most mutated genes across a cohort of 224 TCGA samples.

### 2.8 Statistical Analysis

GraphPad Prism (v9.0) and R software (v4.1.3) were used for statistical analysis. The non-parametric Wilcoxon rank-sum test was employed to compare two groups of samples, while the Kruskal-Wallis test was utilized for comparing multiple groups of samples. A *p* < 0.05 was considered to be statistically significant.

## 3 RESULTS

### 3.1 Identification of Lipid Metabolism-related DEGs in HCC

**Fig. (1):**
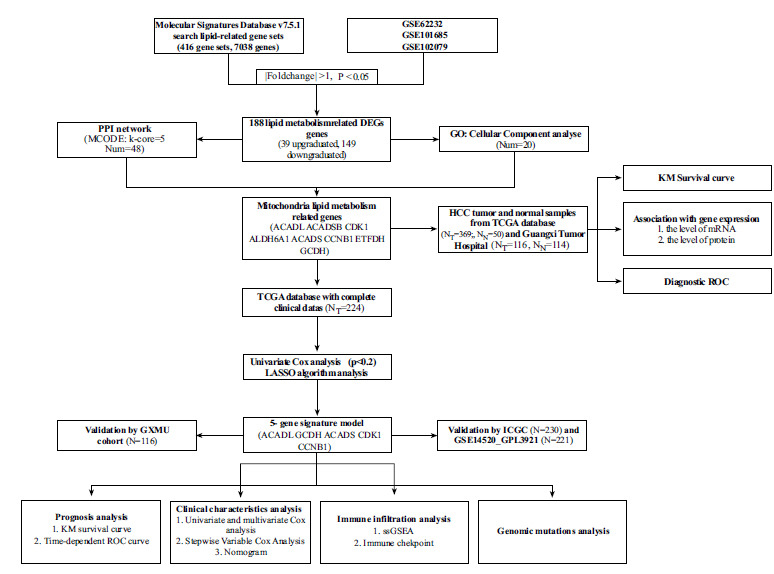
Flow chart of MTLM-related differentially expressed genes (DEGs) identification and validation. **Abbreviations:** PPI, protein-protein interaction; MCODE, Molecular Complex Detection; ROC, receiver operating characteristic; GXMU cohort, The Affiliated Cancer Hospital of Guangxi Medical University cohort; GO, Gene Ontology.

**Fig. (2):**
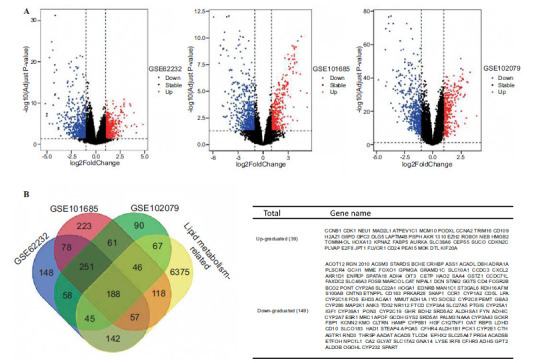
Screening of the dysregulated lipid metabolism-related genes in HCC. (**A**) Volcano plots of DEGs in three GEO cohorts. (**B**) Venn chart showed the number DEGs associated with lipid metabolism-related.

The flowchart demonstrates the identification of MTLM DEGs and the validation process for their diagnostic and prognostic value (Fig. **1**). A total of 7038 lipid metabolism-related genes were obtained from the MSigDB. DEGs identified in the three GEO datasets underwent a rigorous screening process, with a stringent cut-off standard of P value <0.05 and |log2 fold change|>1, as illustrated in Fig. (**2A**). 188 lipid metabolism-related DEGs (39 upregulated and 149 downregulated) were identified across the three GEO datasets (Fig. **2B**).

### 3.2 Extraction of MTLM-related DEGs in HCC

**Fig. (3):**
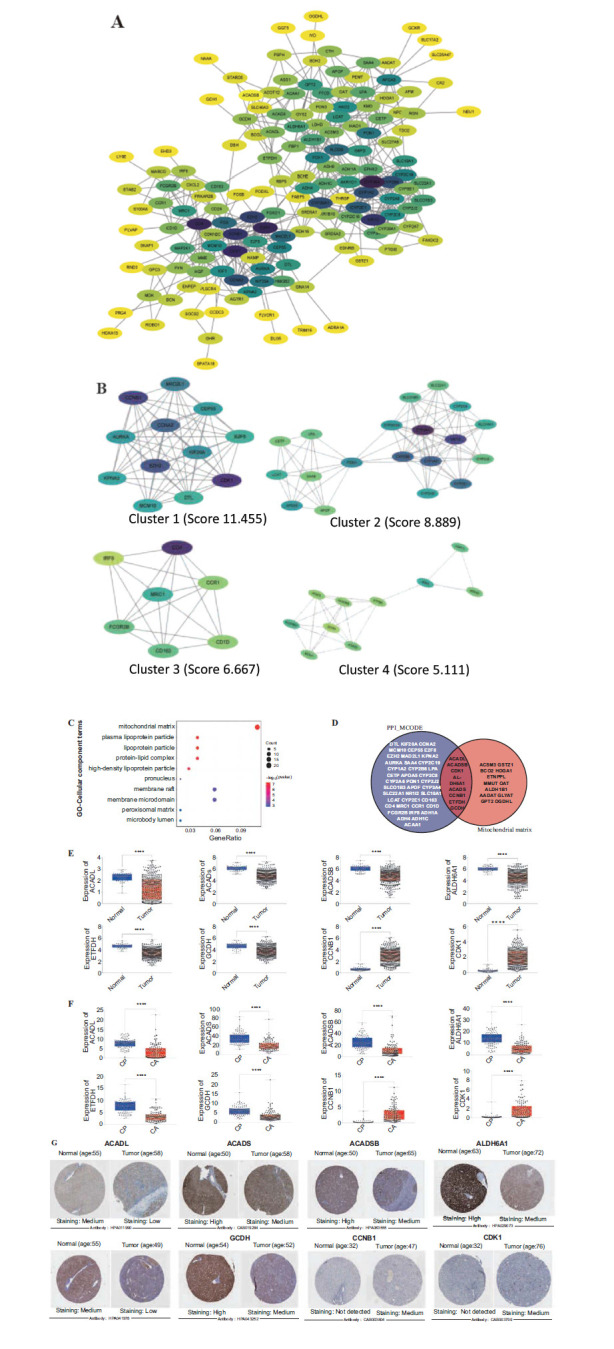
Screening of MTLM-related DEGs. (**A**) Protein interaction network construction of lipid metabolism-related. (**B**) Hub gene clusters were screened with MCODE (PPI_MCODE). (**C**) Functional enrichment analyses of GO-Cellular Component. (**D**) Venn chart diagram representing the number of DEGs associated with MTLM. (**E**) The mRNA expression (FPKM) differences of ACADL, ACADS, ACADSB, ALDH6A1, ETFDH, GCDH, CCNB1, and CDK1 between normal tissue and tumor in TCGA cohort. Normal (n=50), Tumor (n=369). (**F**) The mRNA expression differences of ACADL, ACADS, ACADSB, ALDH6A1, ETFDH, GCDH, CCNB1, and CDK1 between CA tissues and CP tissues in the GXMU cohort. CP (n=114), CA (n=116). (**G**) Human Protein Atlas immunohistochemical analysis of MTLM-related DEGs. (*p < 0.05; **p < 0.01; ***p < 0.001; ****p < 0.0001). **Abbreviatons:** MTLM, Mitochondrial lipid metabolism; CP, para-carcinoma tissues; CA, carcinoma tissues.

The MTLM-related DEGs were screened out through the PPI network and GO analysis. MCODE was used to select 48 hub lipid metabolism-related DEGs from the PPI network (Figs. **3A** and **B**), which GO enrichment analyses revealed that 20 genes were significantly enriched in terms of mitochondrial matrix (Fig. **3C**). Then, a total of 8 MTLM-related DEGs (ACADL (Gene ID: 33), ACADS (Gene ID: 35), ACADSB (Gene ID: 35), ALDH6A1 (Gene ID: 4329), CCNB1 (Gene ID: 891), ETFDH (Gene ID: 891), CDK1 (Gene ID: 983), GCDH (Gene ID: 2639)) were selected *via* taking the intersection set (Fig. **3D**). The difference expression of mRNAs between tumor (or CA: carcinoma) and normal (or CP: para-carcinoma) tissues is illustrated in Figs. (**3E** and **F**). The expression levels of ACADL, ACADS, ACADSB, ALDH6A1, ETFDH, and GCDH were found to be lower in tumor samples compared to normal samples, whereas the expression levels of *CCNB1* and *CDK1* were observed to be higher in tumor than in normal samples. The protein expression depicted in Fig. (**3G**) is consistent with the corresponding gene expression at the transcript level.

### 3.3 Diagnostic Value and KM Survival Analysis of MTLM-related DEGs

**Fig. (4):**
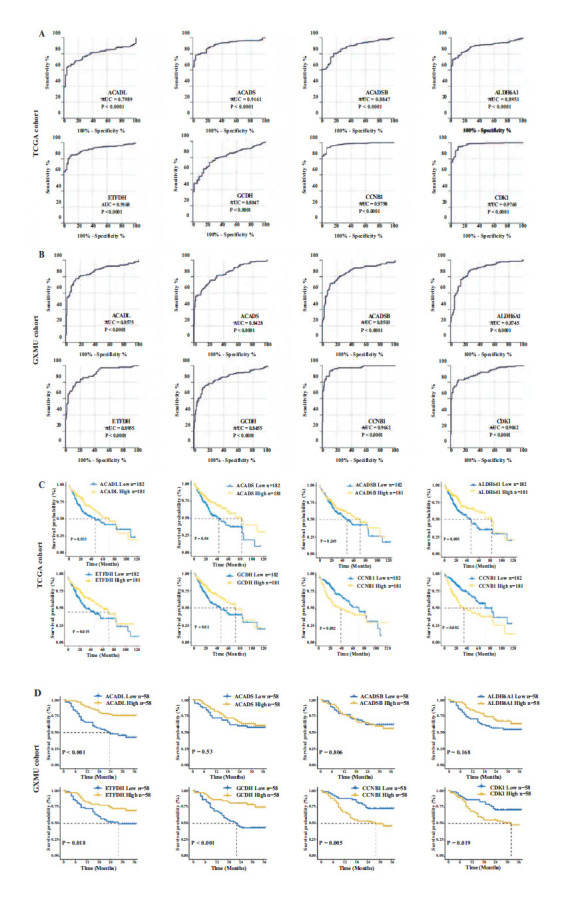
Diagnostic and prognostic value analysis of MTLM genes. The ROC curves evaluate the diagnostic value of MTLM-related DEGs to distinguish between cancer tissues and normal tissues in (**A**) TCGA and (**B**) GXMU cohort. KM survival curves of MTLM-related DEGs in (**C**) TCGA and (**D**) GXMU cohort.

To confirm the diagnostic value of MTLM-related DEGs distinguishing between cancer (or CA) and normal (or CP) tissues, the receiver operating characteristic (ROC) curves were performed by utilizing the data of the TCGA and the GXMU cohort. Based on the findings in Figs. (**4A** and **4B**), these eight genes might serve as potential diagnostic biomarkers for HCC. The association between mRNA expression levels of eight genes and clinical outcomes was evaluated using KM survival curves. The results showed the high expression of CCNB1 and CDK1, and low expression of ACADL, ACADS, ALDH6A1, ETFDH, and GCDH with worse OS. Still, ACADSB did not exhibit significant differences in the TCGA (Fig. **4C**). The survival analysis outcomes obtained from GXMU were similar to TCGA cohort, but no significant differences in ACADS, ACADSB, and ALDH6A1 (Fig. **4D**).

### 3.4 Construction and Evaluation of MTLM-related DEGs Prognostic Signature based on the Training Set

**Fig. (5):**
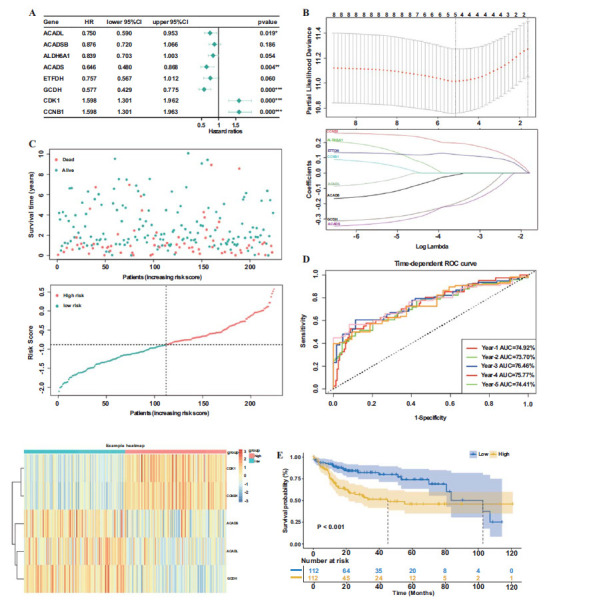
Construction of the optional MTLM gene signature associated with LIHC prognosis in the TCGA database. (A) The forest plots of univariate Cox regression analysis (p < 0.2). (B) Coefficient profiles and Cross-validation for tuning parameter screening in the LASSO regression model. (C) Risk score and survival time distributions, and heat map of gene-expression levels of the MTLM genes signature. (D) ROC curves and AUC values of the risk score model for predicting the 1-, 2-, 3-, 4- and 5-year OS times. (E) Survival curve of the patients in the high and low risk groups. (A higher resolution / colour version of this figure is available in the electronic copy of the article).

Firstly, eight potential OS-associated genes were identified using univariate Cox regression analysis with a p-value cut-off of <0.2 (Fig. **5A**). Subsequently, a prognostic model consisting of five genes was developed through LASSO Cox regression analysis (Fig. **5B**). In this manner, the risk score was computed for each sample: Risk score = expression ACADL*^(-0.0109045518337507)^ + expression GCDH*^(-0.209 35542934212)^ + expression ACADS*^(-0.19513052965704)^ + expression CDK1*^(0.1208468014848)^ + expression CCNB1*^(0.19 5309313766698)^. The median risk score was utilized as a threshold to dichotomize patients into high-risk and low-risk subgroups. Furthermore, the distribution of survival times revealed a positive correlation between higher risk scores and poorer outcomes. The corresponding expression levels of selected genes were determined (Fig. **5C**). The performance of the ROC curve was evaluated for prognoses ranging from 1 to 5 years (Fig. **5D**). The area under the time-dependent ROC curves (AUC) was 74.92%, 73.7%, 76.46%, 75.77% and 74.41%, respectively, for the 1-, 2-, 3-, 4- and 5-year OS times in the TCGA cohort. KM analysis showed that the high-risk group had a significantly shorter OS time than the low-risk group (*p* < 0.001; Fig. **5E**).

### 3.5 Verification of the Optimal MTLM Gene Signature in the External Validation Set

**Fig. (6):**
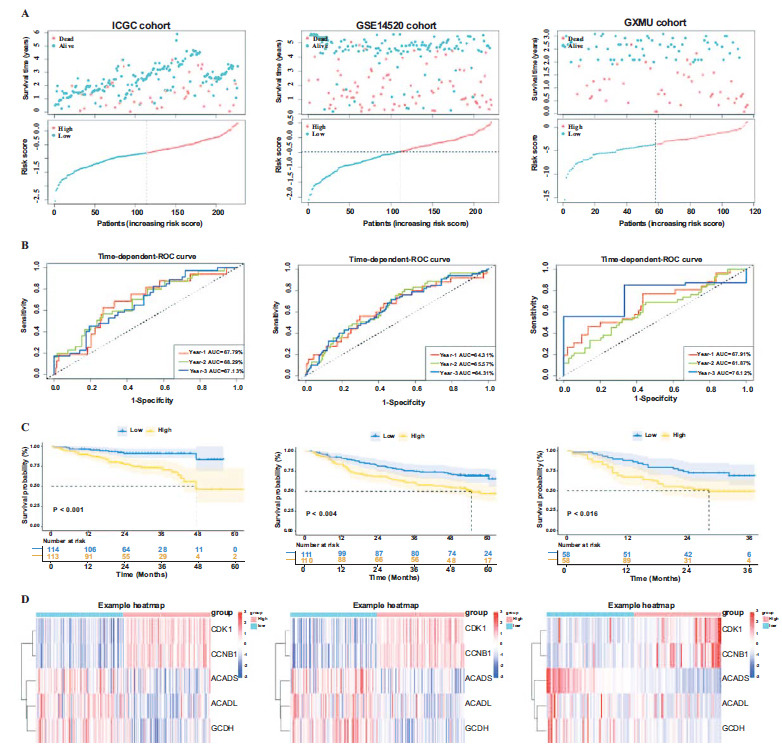
Validation of MTLM gene signature. (**A**) Survival time, survival status (red dots indicate alive, blue dots indicate death), distribution of risk score, (**B**) Time-dependent ROC curve, (**C**) KM survival curve and (**D**) The five genes expression heat maps in ICGC, GSE14520 and the GXMU cohort.

Subsequently, the prognostic model was further validated using external verification cohorts consisting of ICGC, GSE14520-GPL3921 and the GXMU cohort. According to the aforementioned formula, the HCC samples in the validation cohort were categorized into two subgroups (high- or low-risk groups) (Fig. **6A**). ROC analysis indicated that the risk model displayed high accuracy in predicting survival outcomes (Fig. **6B**). Survival analysis revealed that patients classified as high-risk had a significantly worse prognosis (P_ICGC_<0.001; P_GSE14520-GPL3921_=0.004; P_GXMU cohort_=0.016; Fig. **6C**). The heat maps of the five signature genes demonstrated that CDK1 and CCNB1 were significantly upregulated in the high-risk category, whereas *ACADS*, *ACADL*, and *GCDH* were markedly downregulated in the population with elevated risk (Fig. **6D**).

### 3.6 Independent Predicting Ability of the Risk Score and Construction of a Prognostic Nomogram

**Fig. (7):**
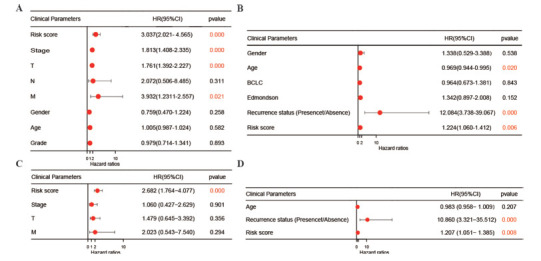
Independent prognosis analysis of the five-gene signature. The result of univariate (**A**, **B**) and multivariate (**C**, **D**) Cox regression performed in the risk score and clinical data based on the TCGA (left) and GXMU (right) cohort.

**Fig. (8):**
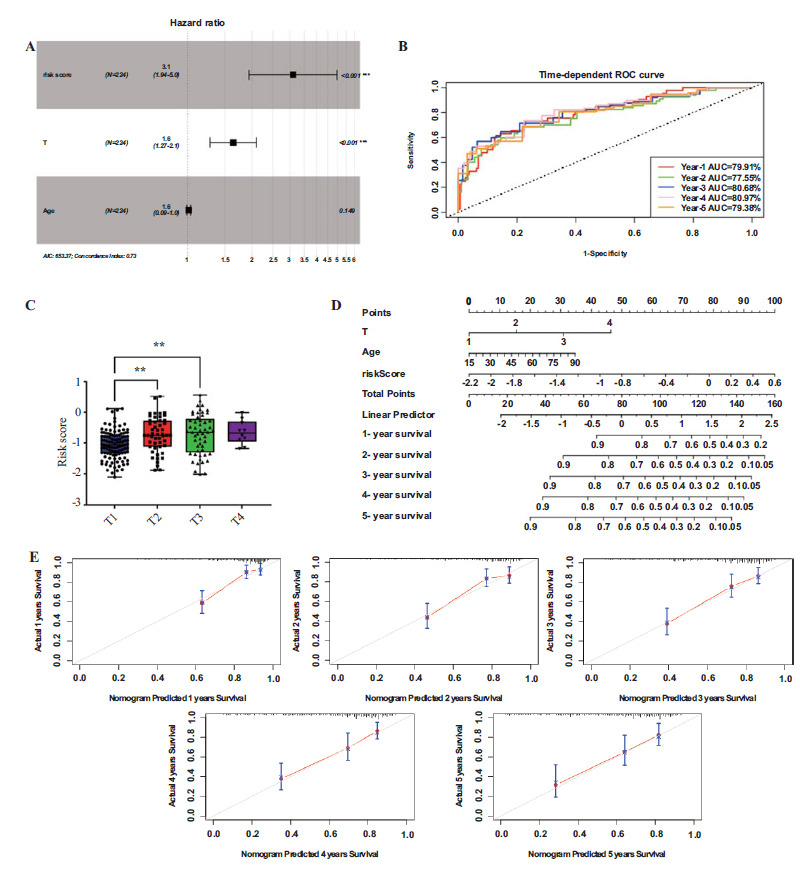
Risk score and clinical features were combined to predict survival. (**A**) The forest plot was drawn by risk score combined with clinical characteristics based on the stepwise variable Cox regression. (**B**) ROC curves and AUC values of the risk score model for predicting the 1-, 2-, 3-, 4- and 5-year OS times. (**C**) Comparison of risk scores of different T stages. (**D**) Nomogram to predict survival probability at 1-, 2-, 3-, 4- and 5-year. (**E**) Calibration curve for the nomogram predicting 1-, 2-, 3-, 4- and 5-year overall survival.

The univariate Cox regression analysis revealed a strong correlation between risk scores with OS in both the TCGA and GXMU cohorts (Figs. **7A** and **B**). Furthermore, multivariate Cox regression analysis confirmed that the risk score remains an independent predictor of OS even after adjusting for other confounding factors (Figs. **7C** and **D**). Moreover, stepwise variable selection was employed to investigate the predictive efficacy of combining risk score and clinical data in the TCGA cohort. The best-fitting Cox proportional hazards model was obtained using (Fig. **8A**). In the TCGA cohort, the AUCs for 1-, 2-, 3-, 4- and 5-year OS times were respectively found to be 79.91%, 77.55%, 80.68%, 80.97%, and 79.38% (Fig. **8B**), which were higher than those obtained using a single risk score (Fig. **5D**). The risk scores of T2 (P=0.0061) and T3 (P=0.0035) expressed significant compare with T1 (Fig. **8C**). The stepwise regression model presented that the integration of the risk score model and conventional clinical parameters yielded superior predictive efficacy. Further, nomogram was developed based on stepwise variable Cox regression analysis to visually demonstrate their prognostic value in predicting overall survival at 1-, 2-, 3-, 4- and 5-year intervals (Fig. **8D**). The concordance index for the training cohort was 0.7171868. Additionally, the nomogram had excellent predictive accuracy for overall survival at 1-5 years, as shown by the calibration curves (Fig. **8E**).

### 3.7 Immune Infiltration and Mutant Genes Analysis According to Prognostic Signature

**Fig. (9):**
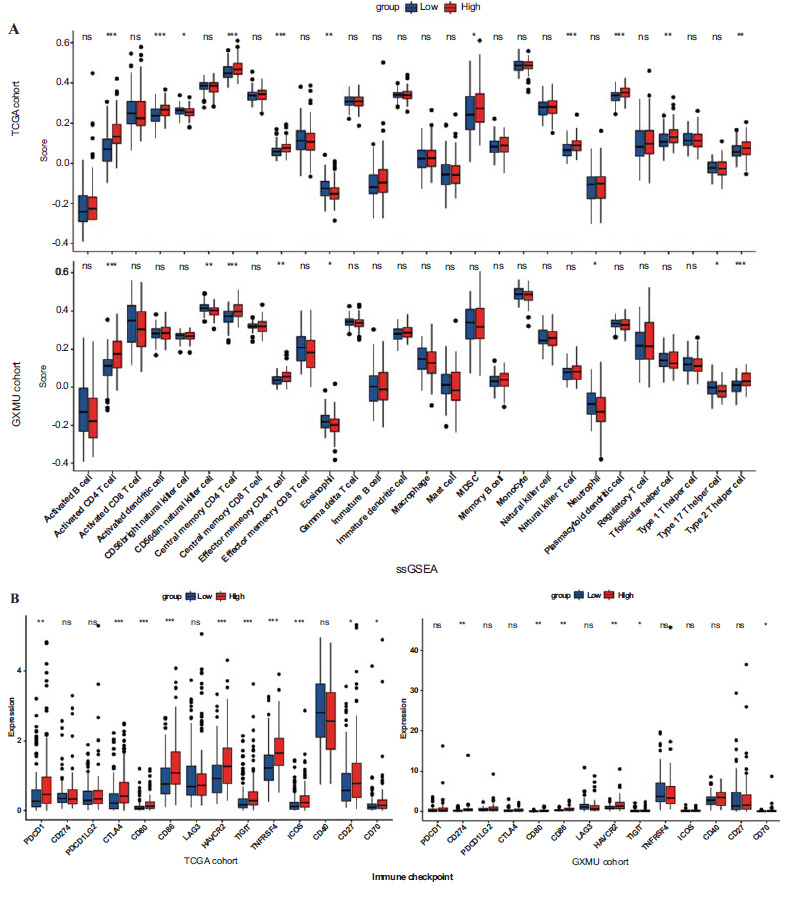
Tumor immune microenvironment and checkpoints analysis in the TCGA database and the GXMU cohort. (**A**) The abundance of immunocytes within tumor microenvironment in high- and low- risk groups. (**B**) Comparison between the two groups for immune checkpoints. (ns, no significance; *p < 0.05; **p < 0.01; ***p < 0.001; ****p < 0.0001).

**Fig. (10):**
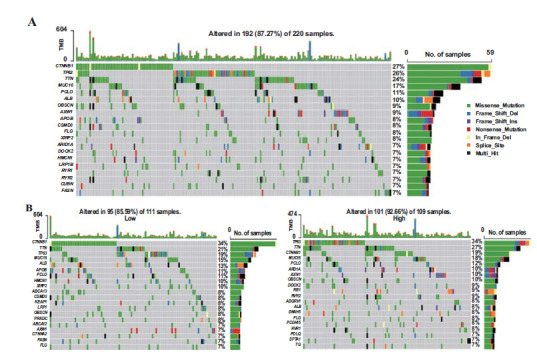
Genomic mutations in the five-gene signature. (**A**) Genomic mutation signature in the patients with HCC from the TCGA database. (**B**) Significantly mutated genes in high and low-risk subgroups. The top 20 mutated genes are listed; the right shows mutation percentage and the top shows the overall mutation rates of different cohorts.

Based on TCGA database and the GXMU cohort, the disparities in the tumor immune microenvironment (TIME) were further assessed across groups of varying risk levels. Fig. (**9**) showed the infiltration abundance of 22 types of immune cells in each group by ssGSEA analysis. The high-risk group exhibited a greater abundance of activated *CD4* T cells, central memory CD4 T cells, effector memory *CD4* T cells, Type 2 T helper cells, as well as a lower abundance of eosinophils compared to the low-risk group in both cohorts. Regarding immune checkpoints, there were significant differential expressions in 10 and 6 checkpoints between the groups of varying risk levels for HCC patients from both the TCGA database and the GXMU cohort (Fig. **9**). The two datasets showed five common immune targets, namely CD80, CD86, HAVCR2, TIGIT and CD70. Finally, gene mutations were further analysed to explore the molecular characteristics of the MTLM subgroups. The top 20 genes with the high mutation rates were subsequently identified (Fig. **10**). Our results demonstrated that the high-risk group exhibited a higher frequency of TP53 mutations (34% *vs*. 19%) and a lower frequency of CTNNB1 mutations (19% *vs*. 34%) compared to the low-risk group (Fig. **10**), underlying potential crosstalk between altered mitochondrial lipid metabolism and TP53/CTNNB1 status. Missense variations were predominant in different subgroups.

## 4 DISCUSSION

HCC remains a formidable challenge to global public health. It is an urgent clinical issue to be addressed for exploring the diagnostic and prognostic value of new therapeutic markers, considering the great heterogeneity of HCC.

Nutrients and oxygen are typically deprived in the cores of solid tumors, and tumor cell proliferation mainly depends on abnormal lipogenesis [19]. Lipids play fundamental biological roles in the body, including energy storage, signaling molecules, and composing cell membrane structures. During cancer development, tumor cells alter their membrane composition to invade other niches, evade cell death mechanisms, and enhance lipid metabolism for energy production and management of oxidative stress [20, 21]. Tumor cells utilize lipid metabolism to modulate the function of stromal and immune cells, thereby creating a favorable microenvironment that confers resistance to therapy and promotes recurrence [22, 23]. Braicu *et al.* conducted a comprehensive lipidomic analysis on serum and tumor tissue samples from patients with high-grade serous ovarian cancer, revealing that lipids belonging to categories such as flagellates, lysophospholipids, phosphatidylcholine, sphingosine and triacylglycerol possess diagnostic and prognostic potential superior to CA-125 as prognostic markers [24]. Therefore, dysregulation of lipid metabolism is considered to be one of the unfavorable prognostic factors in tumors. Recent research has demonstrated that mitochondrial lipids (including phosphatidylethanolamine, cardiolipin, and phosphatidylcholine) regulate mitochondrial function *via* cellular signaling pathways to achieve the desired cellular outcome [25]. During mitochondrial apoptosis, cytochrome c promotes the oxidation of protein and lipid substrates, particularly phosphatidylserine-hydroperoxides, resulting in the accumulation of phosphatidylserine-hydroperoxides and translocation of both cytochrome c and phosphatidylserine-hydroperoxide species to the outer mitochondrial membrane (OMM) [26]. Chu *et al.* discovered that exposure to rotenone and other promitochondrial stimuli results in the externalization of cardiolipin (CL) into the (OMM), subsequently inducing mitophagy [27]. Ceramides activate serine/threonine protein phosphatases, such as *PP1* and *PP2A*, which serve as crucial intracellular effectors in the process of apoptosis [28]. The escape of free radicals, such as ROS and RNS, particularly affects mitochondrial lipid CL oxidation, disrupting energy trade-offs and activating intrinsic death pathways [29]. The relevance of the mitochondrial cholesterol in the response of HCC to mitochondrial-targeting chemotherapy has been demonstrated both *in vitro* and *in vivo* [30]. Thus, it is promising to screen potential diagnostic and prognostic biomarkers for hepatocellular carcinoma from MTLM genes.

In our study, the genes related to MTLM were screened and then explored their diagnostic and prognostic significance in HCC. Eight MTLM-associated genes were screened and demonstrated the diagnostic value by ROC curves. Then, a prognostic signature consisting of five genes (including ACADL, ACADS, GCDH, CCNB1 and CDK1) associated with MTLM was established through univariate Cox and LASSO regression analyses. The external validation sets have revealed that the MTLM five-gene signature exhibits superior predictive power and is an independent prognostic predictor of patient survival. Furthermore, a nomogram combining risk score and clinicopathological characteristics has been constructed, demonstrating high accuracy in estimating OS and recurrence rates, which can effectively guide follow-up care and treatment for individual patients. The Kaplan-Meier survival and diagnostic value curves presented that ACADL, ACADS, ETFDH, GCDH, CCNB1 and CDK1 still had a good predictive performance. However, the *ACADS* did not reveal significant differences in the GXMU cohort, with insufficient sample size being identified as the primary factor.

Furthermore, the ssGSEA analysis has unveiled noteworthy distinctions among subgroups of activated CD4 T cells, central memory CD4 T cells, effector memory CD4 T cells, Type2 T helper cells, and eosinophils. This suggests that MTLM genes may play a crucial role in HCC development through the tumor immune microenvironment. Ma *et al.* reported that the disruption of mitochondrial function by dysregulation of lipid metabolism in non-alcoholic fatty liver disease, leads to selective loss of intrahepatic CD4^+^ T cells and accelerating hepatocarcinogenesis [31]. In addition, regulatory and cytotoxic CD4 T cells have been found to be enriched and clonally amplified in various cancers, including bladder cancer [32, 33]. The CD4 T cell count was unexpectedly higher in the high-risk group than in the low-risk group, according to our study. Further research is needed to investigate how MTLM genes regulate tumor progression through CD4 T cells.

Moreover, mutations of *TP53* and CTNNB1 are the most prevalent molecular abnormalities in Hepatocellular carcinoma, which are mutually exclusive and define high- and low-risk groups characterized by different phenotypes [34]. Activating mutations in CTNNB1 stimulate the Wnt/ βcatenin signaling pathway, which regulates ATP production through the Krebs cycle, oxidative phosphorylation, and fatty acid oxidation [35]. Major events in HCC include alterations of the Wnt/β-catenin signaling pathway [36], P53 pathway, cell cycle mechanisms, and PI3K/AKT/mTOR axis [37], as well as abnormal angiogenesis and epigenetic abnormalities [38]. Our data further support the idea that *TP53* and CTNNB1 mutations collaborate with abnormal MTLM to facilitate cancer progression in HCC, although additional laboratory investigations are warranted in the future. Immunotherapies, particularly immune checkpoint inhibitors (ICIs), are transforming the landscape of cancer management. In recent years, checkpoint inhibitors PD-1, PD-L1, and CTLA-4 have been approved for use in oncological treatment [39, 40]. The combination of the PD-L1 antibody and the anti-VEGFA antibody bevacizumab has been reported to improve OS in HCC when compared with sorafenib [41, 42]. Of note, LAG3, TIM3, TIGIT, HAVCR2, and TIGIT are thought to be targets of co-inhibitory receptors, therapeutic antibodies targeting these receptors are undergoing pre-clinical and clinical testing [32, 43, 44]. Our study indicated that the high-risk group is characterized by the elevated expression levels of CD80, CD86, HAVCR2, TIGIT and CD70. The identification of immune checkpoints provides valuable insights for selecting adjuvant therapies in patients undergoing HCC or radiotherapy. Some data exhibit that the MTLM-associated genes are involved in the occurrence, progression, metastasis, and recurrence of cancer. CCNB1 and CDK1 are key cell cycle molecules that can affect tumor growth and metastasis [45, 46]. Multiple lines of evidence suggest that the p-CDK1-CCNB1 complexes associated with mitochondria trigger phosphorylation of BCL-XL and BCL-2, resulting in the loss of anti-apoptotic, bioenergetic, and metabolic functions in mitochondria governed by BCL-XL/BCL-2 at the endoplasmic reticulum-mitochondria interface [47-49]. ACADL, ACADS and GCDH are member of the acyl-CoA dehydrogenase family. Additionally, the mitochondrial fatty acid beta-oxidation pathway is initiated by ACADS and ACADL catalysis. ACADL was reported to regulate the HCC process by modulating ROS or fatty acid levels [50-52]. ACADS regulates key cellular processes to facilitate tumorigenesis and has been identified as a promising target for cancer therapy by numerous studies [53-55]. GCDH plays a role in amino acid degradation pathways and the TCA cycle [56]. Thus, Guerreiro *et al.* observed that the striatum of GCDH knockout mice exhibited detectable lipid and protein damage, increased production of oxidative stress metabolites, and decreased antioxidant capacity under conditions of lysine overload [57].

## CONCLUSION

In conclusion, this study developed and validated a risk model for HCC based on MTLM-associated genes (ACADL, ACADS, GCDH, CCNB1 and CDK1) through a comprehensive bioinformatic analysis. The risk model as an independent predictor, possesses significant diagnostic and prognostic value for hepatocellular carcinoma (HCC). Furthermore, the stepwise regression model indicated that the risk score combined with conventional clinical parameters had a better predictive effect. Moreover, the nomogram showed a significantly high clinical utility in predicting the 1-, 3-, and 5-year survival probabilities of patients with HCC. The disparities in tumor mutation and immune cell infiltration levels observed between the high and low-risk groups suggest that MTLM-associated genes may exert regulatory effects on the tumor immune microenvironment, thereby providing a novel avenue for future investigations into HCC biomarkers.

## Figures and Tables

**Table 1:** Clinic pathological characteristics of extracted patients with HCC.

**-**	**Clinicopathological Features**	**GXMU Cohort** **(116 Samples)**	**TCGA Cohort** **(224 Samples)**
Age(years)	-	49.50 (42.25 - 60.00)	59.12 (50.75 - 67.44)
Gender	Male	101 (87.1%)	154 (69%)
-	Female	15 (12.9%)	70 (31%)
BCLC	A	58 (50.0%)	-
-	B	32 (27.6%)	-
-	C	26 (22.4%)	-
Edmondson	I	1 (0.9%)	-
-	II	58 (50.0%)	-
-	III	48 (41.4%)	-
-	IV	7 (6%)	-
-	none	2 (1.7%)	-
Pathological stage	Stage I	-	109 (49%)
-	Stage II	-	49 (22%)
-	Stage III	-	62 (28%)
-	Stage IV	-	4 (2%)
Pathological T	T1	-	111 (50%)
-	T2	-	50 (22%)
-	T3	-	53 (24%)
-	T4	-	10 (4%)
Pathological N	N0	-	220 (98%)
-	N1	-	4 (2%)
Pathological M	M0	-	221 (99%)
-	M1	-	3 (1%)
Grade	G1	-	28 (12%)
-	G2	-	98 (44%)
-	G3	-	88 (39%)
-	G4	-	10 (4%)
Fustate	Alive	70 (60.3%)	152 (68%)
-	Dead	46 (39.7%)	72 (32%)
Futime	-	750.00 (450.00 - 960.00)	591 (348.5 - 1235.25)

## Data Availability

The data that support the findings of this study are available from the corresponding author, Feng Wang and Jianhui Yuan, on special request.

## LIST OF ABBREVIATIONS

AUC: Area Under the Curve
GEO: Gene Expression Omnibus
GSEA: Gene Set Enrichment Analysis
GXMU: Guangxi Medical University
HCC: Hepatocellular Carcinoma
ICGC: International Cancer Genome Consortium
MTLM: Mitochondrial Lipid Metabolism
ROC: Receiver Operating Characteristic
TCGA: The Cancer Genome Atlas Program
